# The Rubber Question

**Published:** 1866-11

**Authors:** 


### Editorial.

### THE RUBBER QUESTION.

This subject, as most of our readers are aware, is again before them for consideration.

We had hoped that the annoyance and vexation to the profession, in connection with this subject was at an end, with the expiration of the patent two years ago; but in this we were disappointed, a renewal being obtained about eighteen months ago, for seven years. This fact was unknown to nine-tenths of the profession till within a month or two past.

Why it was not long ago brought to the notice of the profession and some amicable arrangement made, we are not advised. Could the ill success of the American Hard Rubber Company with the Dental profession under the former issue of the patent, have had anything to do with it? Why did not the patentee put the matter into operation with the dentists immediately after the renewal? Why did not those who were before so *very successful*, take hold again? They failed to make it profitable. The patentee had to find new parties, or those unacquainted with the former operations, to purchase and enter upon it now. What will be the result, we shall not now attempt to predict; but our experience warrants us in giving expression to the opinion, that it has usually been found rather a difficult matter for a very few men to compel a very large number of their compeers to do that which the latter believe to be wrong and unjust to themselves, especially for any considerable length of time.

With scarcely an exception, the members of the Dental profession do regard the demands of the Dental Vulcanite Company as unjust and oppressive; and the feeling is, resistance, or non-compliance with such demands.

It is very probable, should the Vulcanite Company come upon the profession in detail, that many will yield to their exactions; but if united resistance is made, it is doubtful whether money enough to pay for the getting will be obtained by the company.

Under the American Hard Rubber Company, simply a license fee was charged, that ranged from $10.00 to $100.00, for a license during the existence of that patent, just as the *bargain* could be made. We know of cases where parties were urged to take license for $20.00, to pay a few dollars in hand, and give a note for the balance, payable on as long time as the purchaser might choose; and even with these very accommodating terms, not more than one in twenty of the profession through the West, who used rubber, purchased license; and in no instance, are we aware, that a prosecution was commenced against any member of the profession in the West for infringement, nor even an injunction asked for; and this, notwithstanding the company was bantered and defied to do so in a great number of instances.

We adduce these things to show, as we think, the weakness of the faith of the former parties operating in this matter. But it may be said, they were merciful—did not wish to be harsh with the profession.

Is it probable, that mercy and kindness were the prevailing feeling with the agents of the American Hard Rubber Company, four or five years ago, when they were taunted, defied and almost kicked out, for merely two or three times presenting their cause.

But, we are asked, why is this opposition by the profession to this matter? It would be difficult in a brief space to present all the grounds of this opposition, but we will mention two or three. And, in the first place we will state, that the spirit of opposition to patents and nostrums is almost as old as the profession itself, and it has grown and become stronger as the profession increases in age; so that now, no one can more effectually bring himself under the ban of the profession than by obtaining a patent for something pertaining to our practice, get up a nostrum, or hold a secret. This opposition was engendered and has grown legitimately.

Dentistry is a specialty of medicine, and entertains, and properly so, a common sympathy, spirit and affinity with it.

The practice of medicine, either in general or specialties, is self-sacrificing; in it men sacrifice ease, comfort, and the procurement of money, to any considerable extent, and subject themselves to continual danger; and all that they may relieve human suffering, even by a very battle with death.

We cannot avoid the belief, that the almost universal verdict would be, such men should not be subjected to all the catchpenny operations of an avaricious money-grasping world; that at least they should have immunity from some of those things which those, whose only object in life is to get money practice upon one another.

Were many of those who practice medicine made rich by it, the case might be viewed differently; but, this very course of life precludes the accumulation of property, except in rare cases. We regard it meaner and quite as criminal to defraud the physician, as to rob the beggar.

A wealthy company now own and have control of the Goodyear patent, so far as it applies to dentistry; and also the patent of John A. Cummings, of Boston, for the application of said process to dental purposes. These patents extend, the former over five years from this time, the latter over fifteen years. The dental profession will not quietly submit to be made mere tax-gatherers for this company during this period, and to be called upon to present a corect statement of business, with the names and residences of patients, and other details too tedious to mention. We doubt not the profession would readily consent to a reasonable adjustment of this matter with the company, perhaps rather upon the ground of avoiding annoyance, than an admission of its justness.

